# Research progress in the application of pressing needle embedding needle in dysphagia after stroke: A review

**DOI:** 10.1097/MD.0000000000038914

**Published:** 2024-07-12

**Authors:** Yanfang Long, Kehui Hu, Yi Zhang

**Affiliations:** a Department of Rehabilitation, Suining Central Hospital, Suining, Sichuan, China.

**Keywords:** dysphagia, pressing needle embedding needle, stroke

## Abstract

Stroke is characterized by “three highs,” and dysphagia is a common dysfunction after stroke. Although some patients can gradually recover from dysphagia with the prolongation of the course of the disease, it is easy to change the prognosis of patients due to complications in the early stage of the disease, and clinical research has shown that pressing needle embedding needles can improve the outcome of patients with dysphagia after stroke. We reviewed the clinical related literature on the treatment of dysphagia after stroke by pressing needle and embedding needle in recent years. The application of press needle embedding can improve swallowing function after stroke, and have more significant effects, which can change the clinical outcome of patients. Pressing needle embedding has significant clinical advantages in the treatment of dysphagia after stroke, which can improve the prognosis of patients.

## 1. Introduction

Dysphagia is a general term for symptoms that refer to eating difficulties or inconvenience caused by lesions in swallowing organs such as the mouth, pharynx, and esophagus. According to Matsumura et al,^[1]^ its incidence rate is approximately 50%. The severity and form of dysphagia vary depending on the location and extent of brain tissue damage. Mild cases may present with coughing when drinking or eating, or recurrent lung infections; severe cases often have complications like electrolyte imbalance, malnutrition, and even choking, which can be life-threatening.^[2]^ Rehabilitation training is the main clinical treatment for poststroke dysphagia. While most patients’ swallowing function improves after training, the speed of recovery and overall effectiveness still require enhancement.

Traditional Chinese medicine posits that stroke-induced dysphagia presents in the throat but stems from the brain, requiring treatment to “unblock meridians and invigorate the brain.” In recent years, acupuncture therapy has made considerable progress in treating poststroke dysphagia, showing significant efficacy. Acupuncture therapy aims to reduce inflammation and pain, dispel wind and cold, unblock meridians, and promote blood circulation and energy flow, balancing Qi and Yin–Yang, enabling orderly physiological activities. Pressing needle burying needle therapy, an external TCM acupuncture technique, integrates traditional acupuncture with modern medical technology, permitting extended needle retention. Pressing needle burying needle therapy can boost metabolism, regulate immunity, and change the microstructure around acupoints, enhancing Qi flow, blood circulation, and meridian unblocking.^[3]^ Through centuries of continuous practice, pressing needle embedding needle has been proven safe, effective, and well-accepted by patients. Below is an overview of recent clinical research advancements.

## 2. Methods

This narrative review was performed by collecting clinical trials, basic research, and reviews on pressing needle embedding needle in dysphagia after stroke.

### 2.1. Search strategy

Literature search was performed in China Biomedical Literature Database (SinoMed), Wanfang Data Knowledge Service Platform (Wanfang), China Journal Full text Database (CNKI), VIP Chinese Journal Service Platform, PubMed, and Web of Science databases from the date of database inception through January 2023. The search included the following keywords: pressing needle embedding needle, press needle embedding, pressing and burying needles, stroke, and dysphagia.

### 2.2. Study selection

We searched for relevant articles and selected articles for further reading based on the title and abstract, all included articles were carefully discussed in the present review. This narrative review does not require ethical approval, because no human/patient data were used.

## 3. The pathogenesis of swallowing disorders after stroke

### 3.1. From the perspective of traditional Chinese medicine

In traditional medicine, poststroke dysphagia is categorized under disease names like “stroke,” “aphasia,” and “throat paralysis” according to its primary symptoms. The “Golden Chamber” states, “when evil invades the organs, the tongue becomes difficult to speak, and saliva is expelled,” indicating that poststroke dysphagia belongs to the category of stroke affecting internal organs. The “Copper Man” mentions, “the mouth is tightly closed, the root of the tongue is constricted, making swallowing difficult,” illustrating that damage to the mouth and tongue organs results in dysphagia. Stroke primarily affects the brain but also involves the heart, liver, spleen, and kidneys. Li Shizhen in “Compendium of Materia Medica” considers the brain to be the residence of the primordial spirit. Building on this, modern physician Shi Xuemin proposed that “the orifices close, the spirit hides, and the spirit does not guide Qi,” indicating that the brain is where the spirit resides. Stroke onset obscures the clear orifices, which in the throat manifests as loss of control over its opening and closing.^[4]^ Texts like “Wind Tongue Strong Unable to Speak Hou” and “Lingshu” indicate a direct or indirect connection between the circulation of the twelve meridians and the throat. Imbalances of Yin and Yang in the body, Qi and blood imbalance, and meridian obstruction due to factors such as wind, fire, phlegm, stasis, and deficiency. When the orifices of the throat are obstructed, swallowing difficulties arise. Following the principle of “where the meridians pass, the treatment reaches,” acupuncture is administered to the relevant acupoints of the tongue and pharyngeal muscles. The sustained and stable stimulation generated can activate the connection between the tongue and the meridians. Regulating the Qi and blood of the meridians, promoting the smooth flow of orifices, restoring the balance of Yin and Yang of the organs, and facilitating the recovery of swallowing function.

### 3.2. From the perspective of modern medicine

Swallowing movement is a complex, multifunctional process with tight coordination among various regions. Impairment of the cerebral cortex, brainstem, and various cranial nerve regions can lead to the occurrence of swallowing difficulties. Particularly common are multiple lesions in the cerebral cortex, brainstem, cerebellum, and thalamus. It is closely associated with cerebral microcirculation disorders and apoptosis of brain neurons.

#### 3.2.1. Cortical and subcortical regions of the brain

The anterior lateral area of both cerebral hemispheres is where the swallowing cortical center is located, with functions of initiating swallowing and regulating the oral-pharyngeal stage. The swallowing central pathway is bilateral. Previous theories suggested that unilateral cranial damage had minimal impact on swallowing function. However, It was found that both hemispheres of the brain have regions affecting swallowing function, and the dominant hemisphere for swallowing is uncertain by Zhong et al.^[5]^ When the dominant hemisphere for swallowing is damaged, swallowing disorders become more severe. Existing research has found that cortical structures related to swallowing are precisely located in areas such as the primary sensory motor area, premotor area, or supplementary motor area, which may participate in integrating sensory motor information during swallowing and forming the concept of swallowing movement.^[6,7]^ Subcortical regions of the brain have roles in initiating, correcting, and inhibiting swallowing actions. Studies^[8]^ have shown that damage to the left subcortical cortex of the brain can easily lead to dysfunction of the pharynx, resulting in increased incidence of aspiration and choking, as well as prolonged swallowing cycles. Damage to the radiating crown portion of the subcortical white matter can hinder the transmission of swallowing cortical information, leading to abnormal release of neurotransmitters in the swallowing center and causing swallowing disorders. Lesions in the subcortical internal capsule can also lead to imbalances in swallowing movements and swallowing disorders.^[9]^

#### 3.2.2. Brainstem and medulla oblongata

Existing research^[10]^ has found that the occurrence of swallowing disorders is associated with degenerative pathological changes in the motor and sensory central nervous system of the brainstem. The main pathological feature is the formation of neuronal vacuoles in areas such as the tail nucleus of the brainstem and the rostral end of the medulla oblongata at the level of the fourth ventricle. The swallowing central pattern generator is located in the medulla oblongata and can initiate or organize swallowing movement sequences, including the solitary tract nucleus-dorsal swallowing group and the ventral swallowing group on the ventrolateral medulla. The central pattern generator controls the timing and sequence of swallowing processes, integrates sensory stimulus information, and the incoming and outgoing processes of the spinal cord’s anterior horn.^[11]^ Swallowing function is regulated by the swallowing-related nerves in the nucleus ambiguus and medulla oblongata. It is now recognized that damage to the brain tissue in the brainstem and medulla oblongata can lead to excitatory or inhibitory rhythm disorders in the neural circuits of swallowing activities, resulting in motor disorders of swallowing movements, prolonged swallowing time, and abnormal sequence and rhythm.

#### 3.2.3. Cerebellum and extrapyramidal system

The cerebellum plays an important role in assisting the coordination of cortical and brainstem swallowing physiological activities. It activates the primary motor cortex for swallowing through the “cerebellum-thalamus-cerebral cortex” neural circuit, participates in feedforward and feedback control, and regulates the excitability of swallowing-related muscles.^[12]^ Damage to the cerebellum leads to impaired motor conduction pathways, causing upper motor neuron paralysis and resulting in the occurrence of swallowing disorders.^[13]^

## 4. The mechanism of action of pressing needle embedding needle

Pressing needle embedding needles belong to a type of intradermal needle and are a development of ancient needle retention methods. When used, the needle is inserted under the skin and fixed with adhesive tape, theoretically providing continuous (24 hour) and stable needle stimulation to the skin and meridians through intradermal needling and embedding treatment. The mechanism of improving swallowing disorders mainly involves discussions from both the 12 cutaneous regions theory in traditional Chinese acupuncture and the neurology theory in modern medicine. In terms of traditional Chinese medicine mechanism, after the needle is embedded under the skin, it exerts its effects through the cutaneous regions and meridians to regulate the meridians. Moreover, leaving the needle in for a long time increases the total stimulation, prolongs the effect of needling, and achieves sustained therapeutic results.^[14]^ “Ling Shu” states: “Examine the defense Qi, which is the mother of all diseases; adjust its deficiency and excess, and the deficiency and excess will be corrected.” It is believed that intradermal needling can stimulate the superficial part of the human body, regulate the defensive Qi, stimulate the defensive Qi of the body, and achieve the purpose of supporting the right and eliminating the evil.^[15]^ In terms of modern medical mechanism, on the one hand, pressing needle embedding needle can strongly stimulate the intradermal nerve tissues of the body, dilate brain blood vessels, enhance cerebral blood flow, increase the excitability of brain cells, promote the reconstruction of swallowing reflexes through the regulatory effects on the central nervous system, and thus promote the recovery of swallowing function. On the other hand, local acupuncture points for pressing needle embedding needle can directly and continuously stimulate the swallowing muscle group, induce muscle contraction, and thus improve blood circulation in the throat and other local organs.^[16]^

Acupoints commonly used for acupuncture treatment include: Lianquan, Jia Lianquan, Yifeng, Jianji 3, 4, 5, Ashi point, etc. Lianquan point belongs to the Ren Meridian, it is the intersection of Yin Wei and Ren Meridian. According to “The Great Compendium of Acupuncture and Moxibustion,” it is recorded that it “treats swelling under the tongue, difficulty in speaking, tongue root contraction, and inability to eat, with saliva flowing vertically,” therefore stimulating Lianquan point can treat stroke, tongue stiffness, and aphasia.^[17]^ It was studied that the acupoint selection rules for acupuncture treatment of dysphagia after stroke, and the results showed that Lianquan point is the core acupoint for treating dysphagia by Wang et al.^[18]^ Yifeng point is the meeting point of the Hand and Foot Shaoyang Meridians. Stimulation of this point is used to treat inability to speak, facial paralysis, and facial swelling. Both Yifeng and Lianquan points are important acupoints in the throat area. The deep part of both points includes tongue muscles, sublingual muscles, and hypoglossal nerves. Combined stimulation can enhance the contraction of swallowing muscles,^[19]^ regulate the autonomic nervous function, promote the reestablishment of reflexes related to swallowing, awaken damaged nerve cells, accelerate the repair of the swallowing reflex arc, and coordinate and improve swallowing movements.^[20]^ Jianji points are located adjacent to the Du Meridian and the Bladder Meridian in the lumbar region. They are commonly used in clinical practice to treat diseases related to the nervous system, and because they are close to the medullary swallowing reflex center, direct stimulation can facilitate swallowing.^[21]^ Ashi point is a temporary acupoint. Traditional Chinese medicine believes that Ashi point is caused by the obstruction of Qi and blood in the meridians of the diseased area. Stimulating this point can promote the dredging of meridians.^[22]^

## 5. Clinical application advantages

Long-term needle retention: Intradermal needles, made of stainless steel wire, with a straight needle body and handle, are often inserted into acupoints that do not affect limb movement, making them suitable for long-term placement in the body for treatment.^[23]^

Simple method: Few insertion points for buried needles, easy to remember, and simple operation methods not restricted by time, location, environment, etc, and no need for patients to undress or remove belts.

Safe, painless, and without side effects: Needles are only retained in the skin, avoiding large blood vessels and nerves, and no important organs, thus low risk, and rarely causing needle fainting. During the buried needle treatment process, there may be slight pain when the needle tip pierces the skin, but it is painless during insertion, making it easily accepted by patients.

The number of treatments is relatively reduced compared to traditional acupuncture, which saves materials and reduces follow-up visits.

Does not affect patients’ daily activities, can be combined with activities such as chewing and swallowing, can be effectively combined with swallowing function training at any time, enhance Qi circulation, promote blood circulation, promote swallowing disorder recovery.

## 6. Common clinical combinations of other therapies

### 6.1. Combined swallowing rehabilitation training

Early rehabilitation training can improve the stiffness of the local tongue muscles in patients with swallowing disorders after stroke, and can also reflexively stimulate the central nervous system, thereby increasing the sensitivity of the neural network.^[24]^ Jiang et al^[25]^ applied swallowing rehabilitation training combined with intradermal needle embedding treatment in 120 patients with swallowing disorders after stroke. After treatment, the number of cases with recovery, improvement, and ineffectiveness of swallowing function in the rehabilitation training combined with intradermal needle embedding group were 19, 32, and 9 respectively, with a treatment effectiveness rate of 85.00%. The number of cases in the pure swallowing rehabilitation training group was 11, 24, and 25 respectively, with a treatment effectiveness rate of 58.33%. Zheng et al^[26]^ applied swallowing rehabilitation training combined with intradermal needle embedding treatment in 118 patients with swallowing disorders after stroke. The experimental results showed that the degree of decrease in the Standard Swallowing Function Assessment Scale score and the volume-viscosity swallow test score, as well as the degree of increase in aerum albumin and total protein, and the activation frequency of the frontal and parietal lobes, were all superior to those in the pure swallowing rehabilitation training group, and the overall effective rate was significantly improved.

### 6.2. Combined neuromuscular electrical stimulation

Neuromuscular electrical stimulation therapy for dysphagia adopts a passive exercise approach, where the low-frequency current generated during treatment acts on the motor endplates, causing passive contraction of swallowing-related muscles, improving muscle strength and coordination of swallowing muscles, thereby assisting in improving swallowing function. Sun et al^[27,28]^ applied neuromuscular electrical stimulation combined with intradermal needle embedding therapy to treat 80 patients with dysphagia after stroke. After treatment, the swallowing function scores and the maximum surface electromyography amplitudes of the genioglossus muscle group in 3 states (relaxation, dry swallowing, and swallowing water) were superior in the neuromuscular electrical stimulation combined with intradermal needle embedding group compared to the pure neuromuscular electrical stimulation group. Xu et al^[29]^ applied neuromuscular electrical stimulation combined with intradermal needle embedding therapy to treat 60 patients with dysphagia after stroke. After treatment, the overall effective rate was 95.0% in the neuromuscular electrical stimulation combined with intradermal needle embedding group, 90.0% in the neuromuscular electrical stimulation combined with electroacupuncture group, and 60.0% in the pure neuromuscular electrical stimulation group. The results showed that intradermal needle embedding combined with neuromuscular electrical stimulation is an effective method for treating dysphagia after stroke.

### 6.3. Combined acupuncture

Wang and Xu^[30]^ randomly divided 60 patients with poststroke oropharyngeal dysphagia into treatment and control groups, with 30 cases in each group. The control group received routine internal medical interventions and acupuncture therapy for awakening the mind, while the treatment group received acupuncture therapy at facial acupoints including Dicang, Jiache, Xiaguan, Quanliao, Qianzheng, and Chengjiang, in addition to the interventions received by the control group. After treatment, the treatment group showed significantly better scores in oral function, swallowing disorder specific quality of life scale scores, and the water swallow test grade compared to the control group. The results indicate that acupuncture therapy combined with buried needle therapy can significantly improve the clinical symptoms and quality of life of patients with poststroke dysphagia. The combination of buried needle therapy and acupuncture can compensate for the shortcomings of conventional acupuncture therapy, prolong the therapeutic effect, and promote the recovery of dysphagia.

### 6.4. Combined traditional Chinese medicine

Patients with dysphagia after stroke may suffer from deficiency of liver and kidney, disharmony between the liver and spleen, leading to deficiency of Qi and blood, resulting in phlegm obstruction and blood stasis. Therefore, traditional Chinese medicine can be used to tonify the liver and kidney, and promote blood circulation to relieve obstruction. Fan et al^[31]^ divided 164 patients with dysphagia after stroke into control group and acupuncture-medicine combination group, with 82 cases in each group. Both groups of patients received routine rehabilitation training. The control group received acupuncture–moxibustion therapy on this basis, while the acupuncture-medicine combination group received acupuncture–moxibustion therapy combined with kidney-nourishing and blood-vessel-opening decoction. After treatment, there were no significant adverse reactions in both groups. The total effective rate of the acupuncture-medicine combination group was 92.7% (76/82), while that of the control group was 81.7% (67/82). The acupuncture-medicine combination group showed significant improvements in the water swallow test results, swallowing function scores, and swallowing disorder specific quality of life scale scores compared to the control group. The results indicate that acupuncture–moxibustion therapy combined with kidney-nourishing and blood-vessel-opening decoction can more effectively improve dysphagia after stroke and improve patients’ quality of life.

### 6.5. Combined muscle patch

Muscle patch has the advantages of convenient operation, no side effects, and good treatment experience, and has good clinical application value. Yu et al^[32]^ divided 90 patients with dysphagia after stroke into muscle patch group, white patch group, and blank control group, with 30 cases in each group. All 3 groups of patients received routine rehabilitation treatment. The muscle patch group received muscle patch and acupuncture–moxibustion therapy, the white patch group received white patch and acupuncture–moxibustion therapy, and the blank control group received acupuncture–moxibustion therapy. After treatment, the muscle patch group showed significant improvement in swallowing function compared to the white patch group and blank control group. The results indicate that the combination of muscle patch and acupuncture–moxibustion therapy is more effective in treating dysphagia after stroke.

## 7. Conclusion

In summary, most existing studies have shown that the application of pressing needle embedding needle can effectively improve swallowing dysfunction in patients after stroke. The combination of pressing needle embedding needle with swallowing rehabilitation training, neuromuscular electrical stimulation, acupuncture, traditional Chinese medicine, muscle patch, and other methods for treating poststroke dysphagia can significantly improve patients’ swallowing ability and thus improve their prognosis. However, current clinical research also faces certain problems, such as generally small sample sizes, inconsistent selection of disease duration, inconsistent intervention periods, and evaluation criteria mostly based on objective scales, lack of long-term efficacy follow-up, and so on. Therefore, in the future, emphasis should be placed on standardizing the treatment guidelines for pressing needle embedding needle, adopting a multidimensional evaluation system, integrating subjective and objective measures, conducting high-quality, multicenter randomized controlled trials or cohort studies to assess the clinical efficacy of pressing needle embedding needle, providing strong evidence-based medicine support for the treatment of poststroke dysphagia with pressing needle embedding needle, making the therapy an effective means of treating this disease, and enhancing the clinical application value of traditional medicine.

## Author contributions

**Conceptualization:** Yanfang Long, Kehui Hu.

**Formal analysis:** Yanfang Long, Yi Zhang.

**Investigation:** Yanfang Long.

**Methodology:** Yi Zhang.

**Writing – original draft:** Yanfang Long.

**Writing – review & editing:** Yanfang Long.
